# Evaluation of the Effects of Different Sample Collection Strategies on DNA/RNA Co-Analysis of Forensic Stains

**DOI:** 10.3390/genes13060983

**Published:** 2022-05-30

**Authors:** Daniela Lacerenza, Giorgio Caudullo, Elena Chierto, Serena Aneli, Giancarlo Di Vella, Marco Barberis, Samuele Voyron, Paola Berchialla, Carlo Robino

**Affiliations:** 1 Department of Public Health Sciences and Pediatrics, University of Turin, 10126 Turin, Italy; lacerenza.d@gmail.com (D.L.); giorgio.caudullo@live.com (G.C.); elena.chierto@unito.it (E.C.); serena.aneli@unito.it (S.A.); giancarlo.divella@unito.it (G.D.V.); 2 S.C. Medicina Legale, AOU Città della Salute e della Scienza, 10126 Turin, Italy; 3 Laboratory of Molecular Genetics, AOU Città della Salute e della Scienza, 10126 Turin, Italy; marco.barberis@gmail.com; 4 Department of Life Sciences and Systems Biology, University of Turin, 10123 Turin, Italy; samuele.voyron@unito.it; 5 Department of Clinical and Biological Sciences, University of Turin, 10043 Orbassano, Italy; paola.berchialla@unito.it

**Keywords:** trace collection, swab, DNA/RNA co-extraction, STR typing, mRNA profiling

## Abstract

The aim of this study was to evaluate the impact of different moistening agents (RNase-free water, absolute anhydrous ethanol, RNAlater^®^) applied to collection swabs on DNA/RNA retrieval and integrity for capillary electrophoresis applications (STR typing, cell type identification by mRNA profiling). Analyses were conducted on whole blood, luminol-treated diluted blood, saliva, semen, and mock skin stains. The effects of swab storage temperature and the time interval between sample collection and DNA/RNA extraction were also investigated. Water provided significantly higher DNA yields than ethanol in whole blood and semen samples, while ethanol and RNAlater^®^ significantly outperformed water in skin samples, with full STR profiles obtained from over 98% of the skin samples collected with either ethanol or RNAlater^®^, compared to 71% of those collected with water. A significant difference in mRNA profiling success rates was observed in whole blood samples between swabs treated with either ethanol or RNAlater^®^ (100%) and water (37.5%). Longer swab storage times before processing significantly affected mRNA profiling in saliva stains, with the success rate decreasing from 91.7% after 1 day of storage to 25% after 7 days. These results may contribute to the future development of optimal procedures for the collection of different types of biological traces.

## 1. Introduction

Multiplex PCR amplification of short tandem repeat (STR) loci, followed by capillary electrophoresis, now enables forensic investigators to recover DNA profiles from minute biological stains and contact traces (the so-called “trace DNA”) found at crime scenes. The identification of cell types in evidentiary traces is often crucial for crime reconstruction. Several robust protocols for DNA/RNA co-extraction from stains have recently been described and validated for forensic purposes, thus enabling the single pipeline analysis of both STRs and mRNA profiles to identify body fluids [1,2,3,4]. Recent studies showed that the simultaneous extraction of DNA and RNA is an effective approach to identify not only the body fluid present, but also the donor(s) of the stain [3,4].

Trace samples are usually collected by means of swabs moistened with water [5]; however, previous studies have shown that alternative swabbing solutions may improve DNA recovery from trace samples, depending on the type of stains and substrates being tested [6,7]. So far, limited research has been dedicated to the effects of the different moistening agents normally applied to collection swabs for RNA extraction and mRNA profiling. However, in a study on DNA/RNA co-extracted from the palmar surface of the hands and fingers of volunteers using different collection methods (including dry/wet swabs and tape-lifting), it was suggested that water may not be the most suitable medium for RNA recovery and preservation in a forensic context because of the high susceptibility of RNA to hydrolytic breakdown [8].

The quantity and quality of the nucleic acids isolated from stains can also be affected by swab storage temperature and by the time interval between trace collection and processing. Immediate processing of swabs appears to improve DNA recovery [9], but this may not always be feasible in casework due to possible logistical constraints. Normally, collection swabs are either frozen or air-dried before further analysis. Swab storage times before processing can then vary widely, depending on the laboratory backlog [10]. It has been shown that the quantities of DNA recovered from frozen and air-dried swabs can vary significantly according to the nature of the sampled body fluid and the type of swab used [11].

As for RNA, molecular analysis was shown to be feasible, even after extended periods, in dried stains [12,13], as well as in 80-year-old archived frozen tissues [14]. Long-term survival of RNA samples suitable for PCR-based body fluid identification was demonstrated in in vitro studies of artificially degraded RNA [15]. Nevertheless, a loss of RNA integrity after prolonged storage at room temperature was also described, especially in body fluids with high endogenous microbial flora, such as saliva [16].

Several reagents devised to preserve both DNA and RNA during the time interval between biological sample collection and analysis were described in the literature, and are currently commercially available [17,18]. However, there is a dearth of studies regarding their efficiency in forensic staining. In this study, mock stains prepared from forensically relevant body fluids—which would typically require mRNA profiling to determine their cellular origin (whole blood, luminol-treated diluted blood, saliva, semen, skin)—were considered. The final aim was to evaluate the impact of different moistening agents applied to collection swabs on DNA/RNA retrieval and integrity for downstream applications. The effects of the swab storage temperature (room temperature vs. freezing) and of swab storage time before DNA/RNA co-extraction were also investigated.

## 2. Materials and Methods

### 2.1. Preparation of Mock Stains

A set of 120 mock stains was prepared by applying 10 μL of the following body fluids obtained from a single donor (consenting healthy male volunteer, aged 33 years) to clean glass slides: whole blood (*n* = 24), whole blood diluted 1:10 with RNase-free water (*n* = 24), saliva (*n* = 24), and semen (*n* = 24). To complete the experimental set, a series of skin samples (*n* = 24) was obtained by rubbing one thumb/index fingertip of the donor over a designated area of the glass slides for approximately 10 s. Rubbing was always performed at a fixed time interval (1 h) after hand washing.

The stains were stored at room temperature (controlled temperature between 15.5 °C and 24 °C, humidity < 60%) and collected 3 days after deposition. Diluted blood was treated with a luminol solution (Bluestar^®^ Forensic, Bluestar, Monte-Carlo, Monaco) immediately before collection.

### 2.2. Collection of Mock Stains

Stains were collected using sterile rayon swabs (Cultiplast, LP Italiana Spa, Milan, Italy) soaked with 20 μL of moistening agent. For each type of stain, one-third of the replicates were collected with RNase-free water (Qiagen, Hilden, Germany), one third with absolute anhydrous ethanol (Carlo Erba, Milan, Italy), and one third with a commercial RNA-stabilizing solution (RNAlater^®^, Qiagen, Hilden, Germany). For each moistening agent, the total number of treated swabs was *n* = 40.

### 2.3. Swab Storage

Prior to DNA/RNA co-extraction, swabs were either air-dried and stored at room temperature (controlled temperature between 15.5 °C and 24 °C, humidity < 60%) (*n* = 60), or frozen at −20 °C (*n* = 60). Swabs were subdivided into two groups so that, for each combination of stain type and moistening agent, equal numbers of samples were either stored at room temperature or frozen.

Swabs were then processed after 1 (*n* = 60) or 7 (*n* = 60) days of storage. Once again, swabs were subdivided into two groups so that equal numbers of samples, each characterized by a specific combination of stain type/moistening agent/storage condition, were processed after 1 or 7 days.

### 2.4. Isolation and Quantitation of Nucleic Acids

Swabs were placed in a spin basket (Investigator Lyse&Spin Basket Kit, Qiagen, Hilden, Germany) with 345 μL RLT buffer, 5 μL Carrier RNA (4 ng/μL), and 13.8 μL DTT (1 M), and incubated for 3 h at 56 °C. The lysate was then collected by centrifugation at maximum speed and subjected to DNA/RNA co-extraction using the AllPrep DNA/RNA Micro Kit (Qiagen, Hilden, Germany), as described by Lacerenza et al. [8], according to the manufacturer’s protocol. The genomic DNA of the donor of the stains was isolated from a buccal swab, using the ChargeSwitch gDNA Normalized Buccal Cell Kit (Invitrogen, Carlsbad, CA, USA) and the KingFisher mL Magnetic Separator (Thermo Fisher Scientific, Vantaa, Finland).

The human genomic DNA extracted from the stains was quantified with the Plexor^®^ HY System (Promega, Madison, IL, USA) and the CFX96 Touch Real-Time PCR Detection System (Bio-Rad, Hercules, CA, USA). The concentration and the RNA Integrity Number (RIN) [19] of the total RNA isolated from the stains were determined using the RNA 6000 Pico Kit^®^ and the Agilent 2100 Bioanalyzer (Agilent Technologies, Santa Clara, CA, USA).

### 2.5. DNA Typing

STR typing was performed using the AmpFlSTR^®^ Identifiler^®^ Plus Kit (Thermo Fisher Scientific, Waltham, MA, USA). The recommended amount of template DNA (1 ng) was included in the PCR whenever possible. For samples with suboptimal DNA concentrations, the maximum volume of input DNA allowed per reaction (10 µL) was employed. Amplifications were then carried out using the 29-PCR cycle protocol, according to the manufacturer’s instructions. Allele detection was performed by capillary electrophoresis on a 3500 Genetic Analyzer (Thermo Fisher Scientific, Waltham, MA, USA) using the GeneMapper software v5.0 (Thermo Fisher Scientific, Waltham, MA, USA) and an analytical threshold of 50 rfu.

### 2.6. mRNA Profiling and Body Fluid Classification

cDNA was synthesized with the RETROscript Kit with Random Decamers (Ambion) using 10 µL of each RNA extract previously treated with DNase (TURBO DNA free, Ambion, Carlsbad, CA, USA).

Seventeen tissue markers (ALAS2, CD93, HBB: blood; HTN3, STATH: saliva; STATH, BPIFA1: nasal mucosa; KLK3, SEMG1, PRM1: semen; CYP2B7P1, MUC4, MYOZ1: vaginal mucosa; MMP7, MMP10, MMP11: menstrual secretions; CDSN, LCE1C: skin), and two housekeeping genes (ACTB and 18S-rRNA) were simultaneously amplified by multiplex end-point PCR, as described by van den Berge et al. [20]. PCR products were purified with the MinElute PCR Purification kit (Qiagen, Hilden, Germany) and detected on a 3500 Genetic Analyzer (Thermo Fisher Scientific, Waltham, MA, USA), using a 50 cm capillary and POP7. Typing was done with GeneMapper software v5.0 (Thermo Fisher Scientific, Waltham, MA, USA) in comparison to reference purified PCR products for each cell type targeted in the multiplex [21]. For each cDNA sample, three PCR replicates with a range of different cDNA inputs (7, 3.5, 1 µL) were initially performed. An optimal cDNA input (i.e., input showing no saturated peaks caused by overexpression of single tissue markers) of 3.5 µL was identified for all stain types, with the exception of semen (1 µL). A total of four mRNA profiling replicates with optimal cDNA input were obtained for each sample.

The classification of the mRNA profiling results was based on the scoring guidelines introduced by Lindenbergh et al. [22]. In brief, a tissue was classified as “observed” if x ≥ n/2, where x indicates the total number of detected tissue-specific peaks above the analytical threshold, and n is the product of the number of replicates (4) and the number of tissue-specific markers included in the multiplex assay (2–3, depending on the tissue type). An analytical threshold of 50 rfu, instead of 150 rfu, as described in [22], was chosen to compensate for the approximately 2-fold reduction in average peak heights observed in the adopted mRNA profiling assay when POP7 is used instead of POP4 [20].

### 2.7. Statistical Analysis

The post hoc Nemenyi test was used to identify statistically significant differences between continuous variables in comparisons between combined subsets of stain types and experimental conditions (i.e., moistening agent, swab storage temperature, and swab storage time before processing). Fisher’s exact test was employed to compare frequencies. The false discovery rate was used as an adjustment for multiple testing.

Generalized log-γ regression was used to assess which of the experimental conditions had influenced the DNA yields the most, as well as to assess the quality of the mRNA profiling results, measured as the mean peak height of the tissue-specific markers and the housekeeping genes in the PCR replicates (non-detected peaks and peaks below the analytical threshold were regarded as 0 rfu in calculations). Odds ratios (ORs) indicating the differential contributions of the experimental factors to body fluid classification (i.e., “observation” of the expected tissues according to the scoring method) were determined by penalized logistic regression.

All analyses were conducted using R version 3.2 [23].

## 3. Results

### 3.1. DNA and RNA Yields

The median concentrations of human genomic DNA isolated from the stains are given in Table 1. The different moistening agents had significant effects on DNA retrieval within single tissues. Water significantly outperformed ethanol in whole blood and semen stains. In the latter case, RNAlater^®^ was also significantly more effective than ethanol. In skin samples, however, swabs treated with ethanol provided significantly higher DNA yields than those treated with water. As for the swab storage conditions, the only significant differences were found in relation to storage temperature, with freezing improving DNA retrieval in luminol-treated diluted blood and saliva.

The median concentrations of the total RNA isolated from the stains are shown in Table 2. The maximization of RNA retrieval following the use of RNAlater^®^ was observed in all tissues (the only exception being saliva), with differences reaching statistical significance in: whole blood, compared to ethanol; luminol-treated diluted blood and skin, compared to water and ethanol; semen, compared to water. As for the storage conditions, the only statistically significant differences in pairwise comparison resulted from swab storage time before extraction. Indeed, 1-day storage gave better results than 7-day storage in saliva; conversely, an increase in RNA yield was observed in semen stains between 1 and 7 days of storage.

The RIN value could only be determined in 78.3% of the samples. A box-plot representation of the distribution of the RIN values is given in Supporting Information, Figure S1. The median RIN value in the measurable samples was 1.85 [IQR: 1–2.4]. In general, RIN values were constantly < 8, with only 6 samples reaching values ≥ 5.

**Figure 1:**
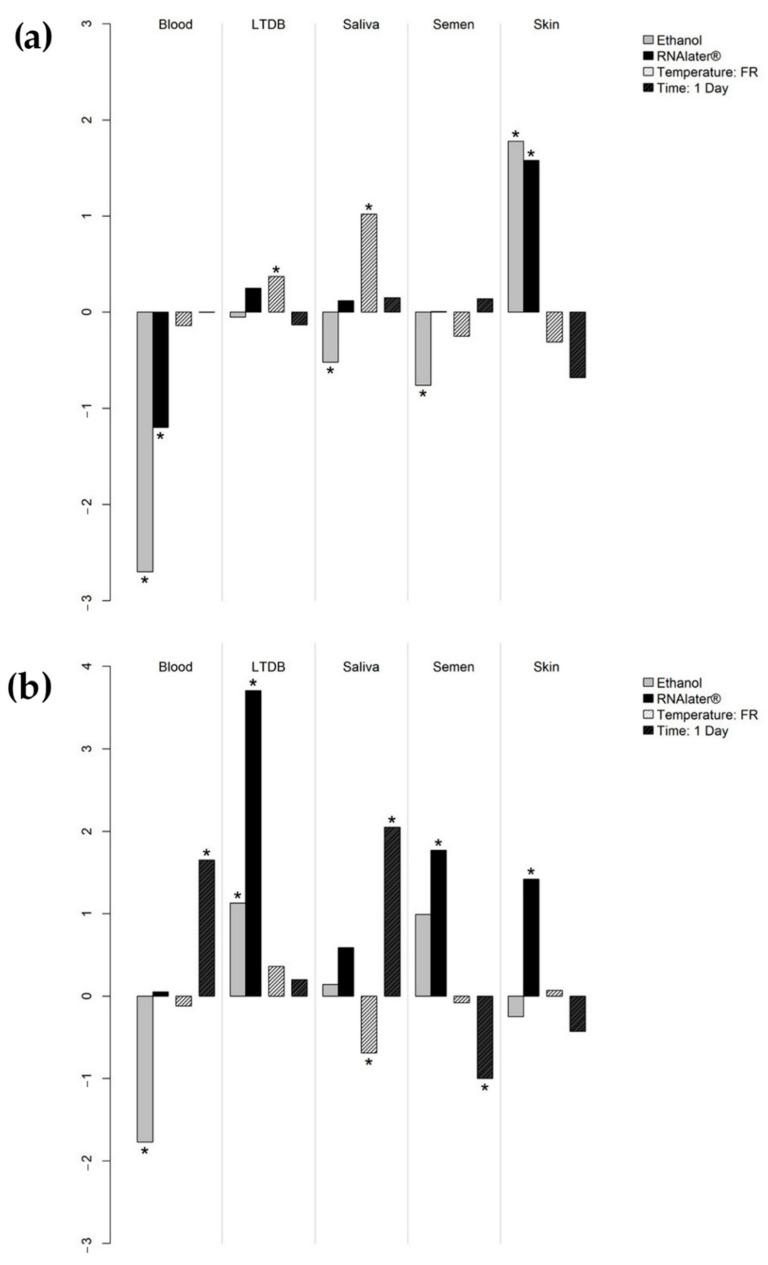
Coefficients on the *y*-axis (log scale) represent the mean increase or decrease in genomic DNA (**a**) and total RNA (**b**) concentrations when different moistening agents, storage temperatures (FR: frozen), and storage times before processing were applied to collection swabs, compared to the standard procedure (i.e., swabs moistened with water and stored at room temperature for 7 days before extraction). Significant *p*-values are marked with an asterisk. LTDB: luminol-treated diluted blood.

The combined effects of the moistening agents and the storage conditions on human genomic DNA (Figure 1a) and total RNA (Figure 1b) retrieval in the different tissues were evaluated by means of generalized log-γ regression, the combination of water, room-temperature storage, and 7-day interval before extraction being considered as the standard procedure; coefficients (mean increase/decrease on a log scale, β) are given in Supporting Information, Table S1.

As for DNA, the results indicated that water was the best moistening agent with almost all tissue types, compared to RNAlater^®^; however, a single statistically significant difference was found in whole blood (β = −1.196, *p* = 0.005). Skin was a remarkable exception, with both ethanol (β = 1.779, *p* = 0.004) and RNAlater^®^ (β = 1.578, *p* = 0.01) significantly outperforming water. The positive effect of freezing on DNA retrieval from the swabs used to collect saliva (β = 1.023, *p* < 0.001) and luminol-treated diluted blood (β = 0.371, *p* = 0.026) was also confirmed. With reference to the influence of moistening agents on RNA, a strikingly different scenario was observed, as water significantly exceeded ethanol in whole blood (β = −1.765, *p* = 0.015), but was significantly outperformed by ethanol in luminol-treated diluted blood (β = 1.133, *p* = 0.002) and by RNAlater^®^ in luminol-treated diluted blood (β = 3.711, *p* < 0.001), semen (β = 1.772, *p* = 0.002), and skin (β = 1.420, *p* < 0.001). Freezing negatively affected the RNA yield from saliva (β = −0.693, *p* = 0.024). Swab storage time before processing had opposite effects on saliva and semen: extraction after 1 day of storage significantly increased RNA retrieval in the first tissue (β = 2.045, *p* < 0.001), but reduced it in the second one (β = −1.002, *p* = 0.019).

### 3.2. DNA Profiling

Irrespective of tissue type, moistening agent and swab storage condition, full STR profiles were obtained from all stains, with the notable exception of the skin samples. Completeness of STR profiles in skin was calculated as the percentage of alleles expected from the donor’s reference profile and effectively observed in the samples. A significant effect of the type of moistening agent applied to the collection swabs was observed, with both ethanol (99.6% ± 0.004% SE) (post hoc Nemenyi test, *p* = 0.006) and RNAlater^®^ (98.7% ± 0.009 SE) (post hoc Nemenyi test, *p* = 0.018) generating, on average, more complete STR profiles than water (71.0% ± 0.052 SE). The negative effect of water on the DNA profile quality of the skin samples was also evident when the peak height ratio (PHR) between alleles of heterozygous genotypes of the donor was considered (Supporting Information, Figure S2a). A sharp increase in allelic imbalance was observed in water-treated swabs at loci with amplicon size >150 bp. In the skin samples collected with the other two moistening agents, the average PHR was constantly >60%, with the sole exception (59%) of the high molecular weight locus CSF1PO in the swabs treated with RNAlater^®^. STR profiles obtained from stain types other than skin had average PHRs >70% at all loci, irrespective of moistening agent (Supporting Information, Figure S2b).

### 3.3. mRNA Profiling

The results of the mRNA profiling experiments are presented in detail in Table 3. The statistically significant negative effect of water on mRNA profiling was evident in whole blood stains, with only 37.5% of the samples being correctly classified, compared to the 100% success rate obtained with ethanol and RNAlater^®^. In saliva stains, both water (37.5% of the samples correctly classified) and ethanol (50.0%) were less performant than RNAlater^®^ (87.5%), but the observed differences did not reach statistical significance. A remarkable difference in mRNA profiling success rates was observed in saliva between swabs processed after 1 day (91.7% of the samples correctly classified) and swabs processed after 7 days (25%) of storage. It is worth noting that 8 out of 10 of the skin samples that had provided incomplete STR typing results were correctly classified as skin in the mRNA profiling experiments.

The differential effects of the moistening agents and of the swab storage conditions on body fluid classification as determined by logistic regression are summarized in Supporting Information Figure S3; ORs are provided in a tabular format in Supporting Information, Table S2. The positive effect of RNAlater^®^ was significant in whole blood (OR 22.350, *p* = 0.01) and saliva (OR 7.857, *p* = 0.048). In the former case, ethanol also significantly outperformed water (OR 22.350, *p* = 0.01). Swab storage time before extraction proved to be a key factor for mRNA profiling success in saliva stains, with the 1-day interval significantly increasing the success rate for body fluid classification, compared to the 7-day interval (OR 20.810, *p* < 0.001). On the contrary, the increase in average RNA yield previously observed in semen stains between 1 and 7 days of storage had no significant effect on mRNA profiling success rate.

**Figure 2:**
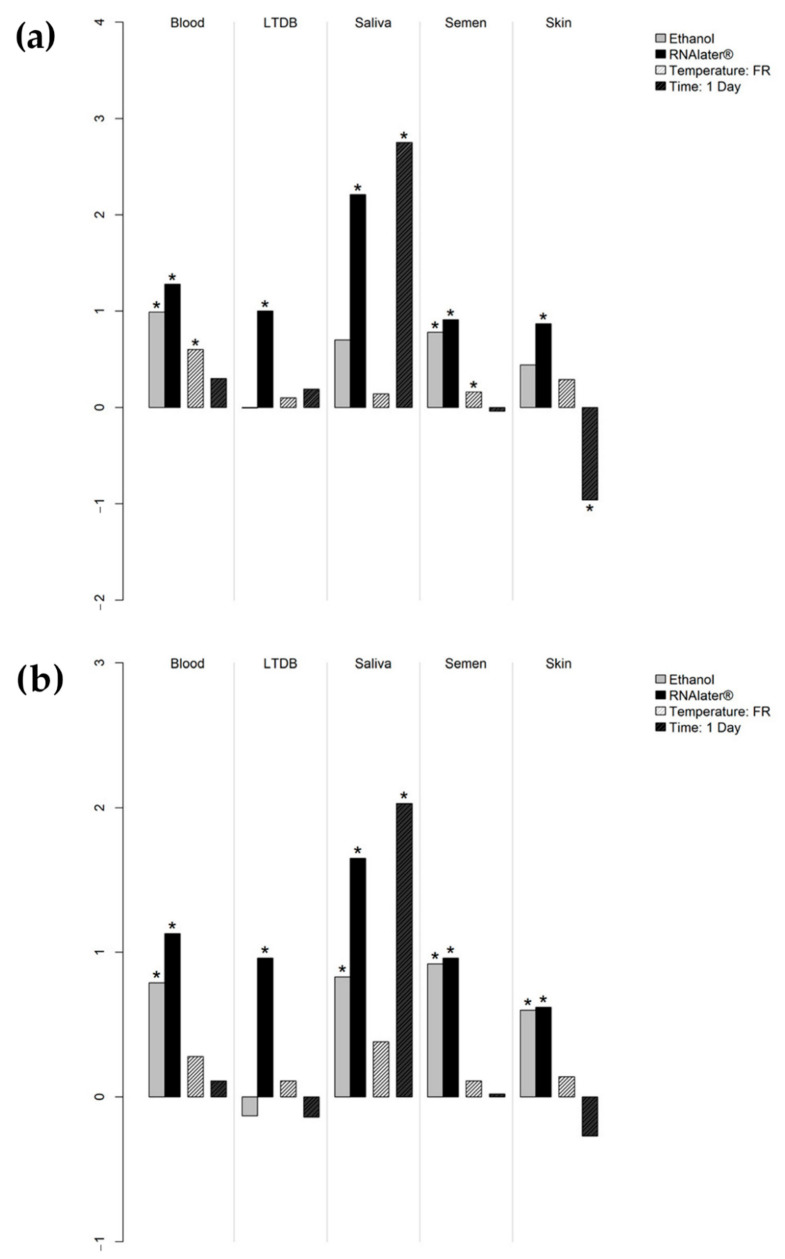
Coefficients on the *y*-axis (log scale) represent the mean increase or decrease in the average peak height (measured in rfu) of the tissue-specific markers (**a**) and the housekeeping genes (**b**) when different moistening agents, storage temperatures (FR: frozen), and storage times before processing were applied to collection swabs, compared to the standard procedure (i.e., swabs moistened with water and stored at room temperature for 7 days before extraction). Significant *p*-values are marked with an asterisk. LTDB: luminol-treated diluted blood.

The influence of the different swabbing procedures on the peak heights was summarized through generalized log-γ regression, the combination of water, room-temperature storage, and 7-day interval before extraction being considered as the standard procedure (Figure 2); coefficients (β) are given in Supporting Information, Table S3. In all tissue types, RNAlater^®^ produced a significant improvement in the amplification signal in both the tissue-specific markers and the housekeeping genes. This is likewise true for ethanol in the collection of whole blood and semen. With reference to the storage temperatures, a significant increase in average peak heights of tissue-specific markers was observed for frozen swabs in whole blood (β = 0.599, *p* = 0.018) and semen (β = 0.155, *p* = 0.049). The amplification signals of both the tissue-specific markers (β = 2.746, *p* < 0.001) and the housekeeping genes (β = 2.029, *p* < 0.001) were significantly enhanced in saliva samples extracted after 1 day of storage, in accordance with the scoring results of mRNA profiling experiments. The opposite was observed in skin samples, but with no significant effect (OR 0.351, *p* = 0.325) on mRNA profiling success rates (Table S2).

## 4. Discussion

Marked tissue-specific differences in DNA/RNA retrieval were observed in relation to the type of moistening agent applied to the swabs. Compared to ethanol, water and RNAlater^®^ proved to be better swabbing solutions for DNA isolation from all tissue types except skin. This could be due to the volatile nature of ethanol, which rapidly evaporated once applied to the collection device and consequently, had a reduced capacity to effectively remove the biological material from the dried stains. In the present experimental set-up, where relatively large volumes of body fluids were used to prepare mock stains (10 μL of whole blood, saliva, semen; 1 μL of whole blood in luminol-treated diluted samples), the lower performance of ethanol did not affect STR typing in terms of DNA profile completeness or peak balance in heterozygous genotypes. However, it can be expected that, when increasingly smaller volumes of body fluids are processed, the low DNA retrieval obtained with ethanol will ultimately have a negative effect on the quality of STR profiling. It should also be considered that, while commonly used by several major laboratories worldwide [24], rayon swabs chosen for this study may not be the best solution for ensuring the highest possible performances in DNA absorption, extraction, and recovery, compared to alternate materials, such as nylon flocked swabs (e.g., FLOQSwabs^®^) [25].

A completely opposite result was observed in skin stains, with both ethanol and RNAlater^®^ significantly outperforming water. The effect was also evident when considering STR profile completeness. Known difficulties in standardizing experiments with replicate skin deposits exist [26]. A limitation of this study was that no preliminary treatment with nucleic acid staining was performed on touched slides to visualize and confirm the amount of transferred skin cells [27]. While care should be taken in interpreting DNA quantitation results obtained from skin samples, the statistically significant differences in DNA profiling results could be partly explained by the lipophilic nature of ethanol, which favored the transfer on the swabs of skin debris containing sweat and oil [28]. The high salinity of RNAlater^®^ may also have promoted the rehydration of sparse cellular material in skin stains, thus improving its retrieval [29]. Possible interference with downstream analysis due to the co-precipitation of the salts contained in RNAlater^®^ was previously described for DNA isolation procedures that include an alcohol-based precipitation step [30]. However, STR profiling experimental results clearly confirmed that the presence of RNAlater^®^ does not interfere with the DNA purification process when a silica-based extraction protocol is used [30].

Another significant factor in improving DNA retrieval in luminol-treated diluted blood and saliva stains was the freezing of swabs after collection. It can be hypothesized that the application of an aqueous solution of luminol immediately before collection slowed down the drying process in swabs, thus favoring the hydrolytic degradation of DNA [31]. In the second case, since saliva is characterized by a high microbial content compared to the other tested body fluids, it was to be expected that quick freezing would improve DNA stability by blocking microbial nuclease activity [31]. It should be noted that rayon swabs have been originally designed to optimize sample retrieval in the context of microbial analyses through an increased moisture-holding capacity, which may explain their reduced efficiency in preserving human DNA when air-dried [32]. The obtained results indicate that, as an alternative to quick freezing, which may not always be possible at the crime scenes, the adoption of swabs equipped with an active drying system is recommended to improve DNA retrieval from luminol-treated and microbe-rich samples, such as saliva [33].

The quantitative assessment of human total RNA in forensic samples is made difficult by the lack of specificity of currently available RNA quantification systems. Results of previous mRNA profiling studies of stains under controlled test conditions indicate that extreme caution is needed when trying to determine the correlation between RNA quantification results and mRNA profiling success [34]. Comparative studies of DNA/RNA co-extraction methods [35,36] also showed that RNA quantification assays (including the RNA 6000 Pico kit and Bioanalyzer used in this study) are subject to a rather large variation in experimental replicates. What was evident from this study was that, as previously suggested [37], the RIN value is of little use as a predictor of mRNA profiling results. Although only 5% of the tested stains had RIN values ≥5, generally considered suitable for downstream molecular applications [38], and none was ≥8, it was possible to obtain correct mRNA profiling results from 82.5% of the tested samples, including 69% of the samples in which the RIN value could not be determined and 65% of those with RIN = 1.

More meaningful considerations on the effects of different sample collection strategies on RNA analysis could be derived from mRNA profiling scoring results and the average peak height of tissue-specific markers and housekeeping genes. The specificity of endpoint PCR primers guarantees that such results strictly reflect human mRNA content in the samples. Moreover, average peak height has been routinely used to evaluate the efficiency of mRNA profiling experiments in mock stains undergoing different types of treatment [16,39]. The use of moistening agents alternative to water improved mRNA profiling results to different extents, depending on the tissue type, with generally higher average peak heights for both tissue-specific markers and housekeeping genes, leading to a significant increase in mRNA profiling success rates in blood samples. The ability of RNAlater^®^ to preserve the RNA molecules is due to its chemical composition based on quaternary ammonium salts, which concur in precipitating RNAses, as well as other solubilized proteins [40]. The fact that, in addition to an RNA-stabilizing solution like RNAlater^®^, ethanol also generally proved to be a more suitable moistening agent than water for collection swabs that were to undergo RNA analysis could be explained by its dehydrating effect. In contrast to DNA, the RNA molecule contains a hydroxyl group (2′-OH) at the 2′ position of the sugar, which allows the RNA molecule to be more easily degraded via hydrolysis than the DNA molecule [41]. The absence of water was shown to reduce RNAse activity and overall RNA degradation in dry stains [13,42]. Although RNAlater^®^ generally performed better than ethanol in terms of mRNA profiling results (average peak height of tissue-specific markers and housekeeping genes), its use during the collection process required great care, as it formed droplets on the tip of the rayon swabs rather than being absorbed by them.

While it is well known that luminol treatment has no appreciable inhibitory effect on subsequent DNA typing [43], very few studies exist in the literature regarding its effect on mRNA profiling. The luminol formulations used in the detection of latent bloodstains are strongly alkaline [44], and it is known that alkali conditions increase the susceptibility of RNA to hydrolysis [45]. Despite this, it was recently reported that stains previously identified by luminol in casework items showed positive results for the three tested blood specific markers HBB, ALAS2, and CD93 in mRNA profiling experiments [46]. Due to the wide array of substances that can cross-react with luminol [47] and to the possible interference of luminol itself with immunochromatographic tests [48], demonstration of the feasibility of mRNA-based confirmatory tests on blood traces detected by luminol chemiluminescence, in agreement with previous reports [46], is particularly relevant for crime scene investigations. The correct identification by mRNA profiling of 80% of the skin samples with incomplete STR profiles was also remarkable, a result that is consistent with previous observations showing that skin mRNA markers may remain detectable, even when DNA profiling efficiency decreases [20].

The most relevant effect of the swab storage conditions on mRNA profiling was the significant decrease in the success rates of body fluid identification following the extension of swab storage time before processing from 1 to 7 days in saliva samples. An early decay of the signal intensity in mRNA profiling (measured as the mean peak heights of the tissue-specific markers STATH and HTN3) was previously observed for saliva stains, in comparison with blood and semen [16]. The present study confirms these results with regard to the mean peak height of tissue-specific markers and housekeeping genes, and overall mRNA profiling success rates, which decreased to 25% after 1 week of storage. The effect is more evident than what was reported in [16], where only stains created from ≤0.5 µL volumes of saliva and stored at room temperature for more than 1 week were affected. The observed rapid decay in mRNA profiling efficiency of saliva samples may be partly due to the type of swab material used in the present study (rayon), which can favor microbial proliferation [32]. According to our results, immediate freezing of the swabs used to collect saliva stains improved mRNA profiling signal intensity and success rates, although not significantly.

## 5. Conclusions

The obtained results confirm that the outcome of forensic DNA/RNA analysis can be greatly influenced, not only by the choice between different types of swabs or by the use of alternative sample collection devices (e.g., tape-lifting) [49], but also by the type of moistening agent applied to swabs. The results also show that the current standard methods for the collection of stains and the storage of collection swabs before processing can affect DNA/RNA profiling results.

This influence appears to be mainly tissue-specific, as in the case of the marked difference in DNA retrieval between skin and non-skin stains observed following the use of ethanol. In casework, especially when dealing with minute stains, it is rarely possible to immediately identify the cellular origin of the biological evidence under investigation. The choice of specific sample collection protocols by crime scene analysts and forensic biology laboratories may therefore depend on various factors [10], e.g., the use of a single method is effective in terms of cost and practicality. However, the development of compact multipurpose kits that include different collection devices (such as those available to police forces for fingerprint detection) [50] and of flexible storage strategies that are dependent on both the presumed origin of the collected stains and the type of molecular analysis to be performed (RNA and/or DNA) might be considered in the future. Meanwhile, further research is necessary to investigate the several variables that may influence DNA/RNA profiling and that were not addressed (or were addressed only to a limited extent) in the present study. These include, among others: wider ranges of sample collection devices (e.g., different swab materials and active fast drying systems), moistening and DNA/RNA preserving agents and substrates (porous/non-porous, sticky/non-sticky, etc.); other forensically relevant body fluids (e.g., menstrual secretion and vaginal mucosa); small stain volumes and extended storage times before processing of sample collection devices.

## Figures and Tables

**Table 1:** Median concentrations of human genomic DNA in experimental samples subdivided by stain type, moistening agent, storage temperature (RT: room temperature), and storage time before processing. The interquartile range (IQR) is given between brackets. *p*-values of the Nemenyi test are shown in the column to the right (ns denotes non-significance).

	Median DNA Concentration (ng/μL)	Post Hoc Test (Nemenyi Test)	
**Blood (*n* = 24)**			
**Moistening Agent**			
Water (*n* = 8)	4.727 [3.680–5.339]	Water vs. Ethanol	*p* < 0.001
Ethanol (*n* = 8)	0.166 [0.120–0.302]	Water vs. RNAlater^®^	ns
RNAlater^®^ (*n* = 8)	1.320 [0.969–1.506]	Ethanol vs. RNAlater^®^	ns
**Storage temperature**			
RT (*n* = 12)	1.106 [0.607–3.180]	RT vs. Frozen	ns
Frozen (*n* = 12)	1.245 [0.308–4.113]		
**Storage time**			
1 day (*n* = 12)	1.105 [0.287–4.025]	1 day vs. 7 days	ns
7 days (*n* = 12)	1.115 [0.737–1.922]		
**Luminol-treated diluted blood (*n* = 24)**			
**Moistening agent**			
Water (*n* = 8)	0.117 [0.063–0.131]	Water vs. Ethanol	ns
Ethanol (*n* = 8)	0.084 [0.065–0.137]	Water vs. RNAlater^®^	ns
RNAlater^®^ (*n* = 8)	0.133 [0.122–0.149]	Ethanol vs. RNAlater^®^	ns
**Storage temperature**			
RT (*n* = 12)	0.095 [0.063–0.128]	RT vs. Frozen	*p* = 0.019
Frozen (*n* = 12)	0.137 [0.103–0.169]		
**Storage time**			
1 day (*n* = 12)	0.103 [0.066–0.141]	1 day vs. 7 days	ns
7 days (*n* = 12)	0.129 [0.103–0.137]		
**Saliva (*n* = 24)**			
**Moistening agent**			
Water (*n* = 8)	0.328 [0.220–0.578]	Water vs. Ethanol	ns
Ethanol (*n* = 8)	0.230 [0.135–0.320]	Water vs. RNAlater^®^	ns
RNAlater^®^ (*n* = 8)	0.373 [0.198–0.542]	Ethanol vs. RNAlater^®^	ns
**Storage temperature**			
RT (*n* = 12)	0.151 [0.127–0.245]	RT vs. Frozen	*p* < 0.001
Frozen (*n* = 12)	0.473 [0.336–0.578]		
**Storage time**			
1 day (*n* = 12)	0.296 [0.203–0.416]	1 day vs. 7 days	ns
7 days (*n* = 12)	0.305 [0.127–0.547]		
**Semen (*n* = 24)**			
**Moistening agent**			
Water (*n* = 8)	23.020 [14.706–31.575]	Water vs. Ethanol	*p* = 0.029
Ethanol (*n* = 8)	9.114 [8.492–12.148]	Water vs. RNAlater^®^	ns
RNAlater^®^ (*n* = 8)	22.770 [17.380–25.110]	Ethanol vs. RNAlater^®^	*p* = 0.022
**Storage temperature**			
RT (*n* = 12)	19.545 [13.635–26.527]	RT vs. Frozen	ns
Frozen (*n* = 12)	14.375 [9.045–20.015]		
**Storage time**			
1 day (*n* = 12)	19.990 [13.164–26.398]	1 day vs. 7 days	ns
7 days (*n* = 12)	14.330 [9.045–19.100]		
**Skin (*n* = 24)**			
**Moistening agent**			
Water (*n* = 8)	0.007 [0.003–0.010]	Water vs. Ethanol	*p* = 0.014
Ethanol (*n* = 8)	0.038 [0.014–0.091]	Water vs. RNAlater^®^	ns
RNAlater^®^ (*n* = 8)	0.020 [0.011–0.059]	Ethanol vs. RNAlater^®^	ns
**Storage temperature**			
RT (*n* = 12)	0.011 [0.008–0.041]	RT vs. Frozen	ns
Frozen (*n* = 12)	0.020 [0.011–0.048]		
**Storage time**			
1 day (*n* = 12)	0.012 [0.008–0.020]	1 day vs. 7 days	ns
7 days (*n* = 12)	0.028 [0.010–0.057]		

**Table 2:** Median concentrations of total RNA in experimental samples subdivided by stain type, moistening agent, storage temperature (RT: room temperature), and storage time before processing. The IQR is given between brackets. *p*-values of the Nemenyi test are shown in the column to the right (ns denotes non-significance).

	Median RNA Concentration (ng/μL)	Post Hoc Test (Nemenyi Test)	
**Blood (*n* = 24)**			
**Moistening Agent**			
Water (*n* = 8)	0.023 [0.014–0.210]	Water vs. Ethanol	ns
Ethanol (*n* = 8)	0.013 [0.009–0.024]	Water vs. RNAlater^®^	ns
RNAlater^®^ (*n* = 8)	0.127 [0.056–0.165]	Ethanol vs. RNAlater^®^	*p* = 0.020
**Storage temperature**			
RT (*n* = 12)	0.029 [0.012–0.120]	RT vs. Frozen	ns
Frozen (*n* = 12)	0.026 [0.015–0.073]		
**Storage time**			
1 day (*n* = 12)	0.078 [0.012–0.032]	1 day vs. 7 days	ns
7 days (*n* = 12)	0.023 [0.013–0.032]		
**Luminol-treated diluted Blood (*n* = 24)**			
**Moistening agent**			
Water (*n* = 8)	0.027 [0.024–0.061]	Water vs. Ethanol	ns
Ethanol (*n* = 8)	0.096 [0.086–0.152]	Water vs. RNAlater^®^	*p* < 0.001
RNAlater^®^ (*n* = 8)	1.669 [0.882–2.268]	Ethanol vs. RNAlater^®^	*p* = 0.047
**Storage temperature**			
RT (*n* = 12)	0.095 [0.042–0.882]	RT vs. Frozen	ns
Frozen (*n* = 12)	0.152 [0.066–0.267]		
**Storage time**			
1 day (*n* = 12)	0.077 [0.041–1.849]	1 day vs. 7 days	ns
7 days (*n* = 12)	0.103 [0.073–0.267]		
**Saliva (*n* = 24)**			
**Moistening agent**			
Water (*n* = 8)	0.155 [0.073–0.230]	Water vs. Ethanol	ns
Ethanol (*n* = 8)	0.103 [0.039–0.178]	Water vs. RNAlater^®^	ns
RNAlater^®^ (*n* = 8)	0.062 [0.041–0.553]	Ethanol vs. RNAlater^®^	ns
**Storage temperature**			
RT (*n* = 12)	0.110 [0.044–0.383]	RT vs. Frozen	ns
Frozen (*n* = 12)	0.094 [0.043–0.187]		
**Storage time**			
1 day (*n* = 12)	0.223 [0.142–0.334]	1 day vs. 7 days	*p* < 0.001
7 days (*n* = 12)	0.040 [0.024–0.047]		
**Semen (*n* = 24)**			
**Moistening agent**			
Water (*n* = 8)	0.555 [0.353–2.788]	Water vs. Ethanol	ns
Ethanol (*n* = 8)	3.278 [1.942–4.146]	Water vs. RNAlater^®^	*p* = 0.039
RNAlater^®^ (*n* = 8)	5.574 [4.696–6.487]	Ethanol vs. RNAlater^®^	ns
**Storage temperature**			
RT (*n* = 12)	4.186 [0.973–6.487]	RT vs. Frozen	ns
Frozen (*n* = 12)	3.278 [1.125–4.878]		
**Storage time**			
1 day (*n* = 12)	1.696 [0.422–4.783]	1 day vs. 7 days	*p* = 0.045
7 days (*n* = 12)	5.005 [3.605–6.701]		
**Skin (*n* = 24)**			
**Moistening agent**			
Water (*n* = 8)	0.111 [0.064–0.128]	Water vs. Ethanol	ns
Ethanol (*n* = 8)	0.109 [0.056–0.141]	Water vs. RNAlater^®^	*p* = 0.004
RNAlater^®^ (*n* = 8)	0.415 [0.364–0.464]	Ethanol vs. RNAlater^®^	*p* = 0.005
**Storage temperature**			
RT (*n* = 12)	0.136 [0.097–0.366]	RT vs. Frozen	ns
Frozen (*n* = 12)	0.193 [0.087–0.364]		
**Storage time**			
1 day (*n* = 12)	0.122 [0.077–0.391]	1 day vs. 7 days	ns
7 days (*n* = 12)	0.171 [0.120–0.360]		

**Table 3:** Percentages of the experimental samples in which the expected tissue was “observed,” according to the adopted scoring method for mRNA profiling, subdivided by stain type, moistening agent, storage temperature, and storage time before processing. *p*-values of Fisher’s test adjusted for multiple testing are shown in the column to the right (ns denotes non-significance).

	Expected Tissue “Observed”	Fisher’s Exact Test	
**Blood (*n* = 24)**			
**Moistening Agent**			
Water (*n* = 8)	37.5%	Water vs. Ethanol	*p* = 0.04
Ethanol (*n* = 8)	100%	Water vs. RNAlater^®^	*p* = 0.04
RNAlater^®^ (*n* = 8)	100%	Ethanol vs. RNAlater^®^	ns
**Storage temperature**			
RT (*n* = 12)	75.0%	RT vs. Frozen	ns
Frozen (*n* = 12)	83.3%		
**Storage time**			
1 day (*n* = 12)	83.3%	1 day vs. 7 days	ns
7 days (*n* = 12)	75.0%		
**Luminol-treated diluted Blood (*n* = 24)**			
**Moistening agent**			
Water (*n* = 8)	100%	Water vs. Ethanol	ns
Ethanol (*n* = 8)	100%	Water vs. RNAlater^®^	ns
RNAlater^®^ (*n* = 8)	87.5%	Ethanol vs. RNAlater^®^	ns
**Storage temperature**			
RT (*n* = 12)	91.7%	RT vs. Frozen	ns
Frozen (*n* = 12)	100%		
**Storage time**			
1 day (*n* = 12)	100%	1 day vs. 7 days	ns
7 days (*n* = 12)	91.7%		
**Saliva (*n* = 24)**			
**Moistening agent**			
Water (*n* = 8)	37.5%	Water vs. Ethanol	ns
Ethanol (*n* = 8)	50%	Water vs. RNAlater^®^	ns
RNAlater^®^ (*n* = 8)	87.5%	Ethanol vs. RNAlater^®^	ns
**Storage temperature**			
RT (*n* = 12)	50%	RT vs. Frozen	ns
Frozen (*n* = 12)	66.7%		
**Storage time**			
1 day (*n* = 12)	91.7%	1 day vs. 7 days	*p* = 0.003
7 days (*n* = 12)	25.0%		
**Semen (*n* = 24)**			
**Moistening agent**			
Water (*n* = 8)	87.5%	Water vs. Ethanol	ns
Ethanol (*n* = 8)	100%	Water vs. RNAlater^®^	ns
RNAlater^®^ (*n* = 8)	100%	Ethanol vs. RNAlater^®^	ns
**Storage temperature**			
RT (*n* = 12)	100%	RT vs. Frozen	ns
Frozen (*n* = 12)	91.7%		
**Storage time**			
1 day (*n* = 12)	100%	1 day vs. 7 days	ns
7 days (*n* = 12)	91.7%		
**Skin (*n* = 24)**			
**Moistening agent**			
Water (*n* = 8)	75.0%	Water vs. Ethanol	ns
Ethanol (*n* = 8)	75.0%	Water vs. RNAlater^®^	ns
RNAlater^®^ (*n* = 8)	100%	Ethanol vs. RNAlater^®^	ns
**Storage temperature**			
RT (*n* = 12)	75%	RT vs. Frozen	ns
Frozen (*n* = 12)	91.7%		
**Storage time**			
1 day (*n* = 12)	75%	1 day vs. 7 days	ns
7 days (*n* = 12)	91.7%		

## Data Availability

The data that support the findings of this study are available from the corresponding author upon reasonable request.

## Supplementary Materials

The following supporting information can be downloaded at: https://www.mdpi.com/article/10.3390/genes13060983/s1, Figure S1: RNA Integrity Number (RIN) values; Figure S2: Average peak height ratio (PHR) in skin and other tissue samples; Figure S3: mRNA profiling results (Odds ratios); Table S1: DNA/RNA concentrations; Table S2: mRNA profiling results (Odds ratios, Confidence Intervals and p-values); Table S3: peak height (rfu) of tissue-specific mRNA markers.
