# Supplementary material for: Evaluation of the Effects of Different Sample Collection Strategies on DNA/RNA Co-Analysis of Forensic Stains
Source: Genes (Basel). 2022 May 30;13(6):983. doi: 10.3390/genes13060983 (PMC9222428; doi:10.3390/genes13060983)
Supplement: Supplementary file 1 [file genes-13-00983-s001.zip › genes-1716677-supplementary.pdf]

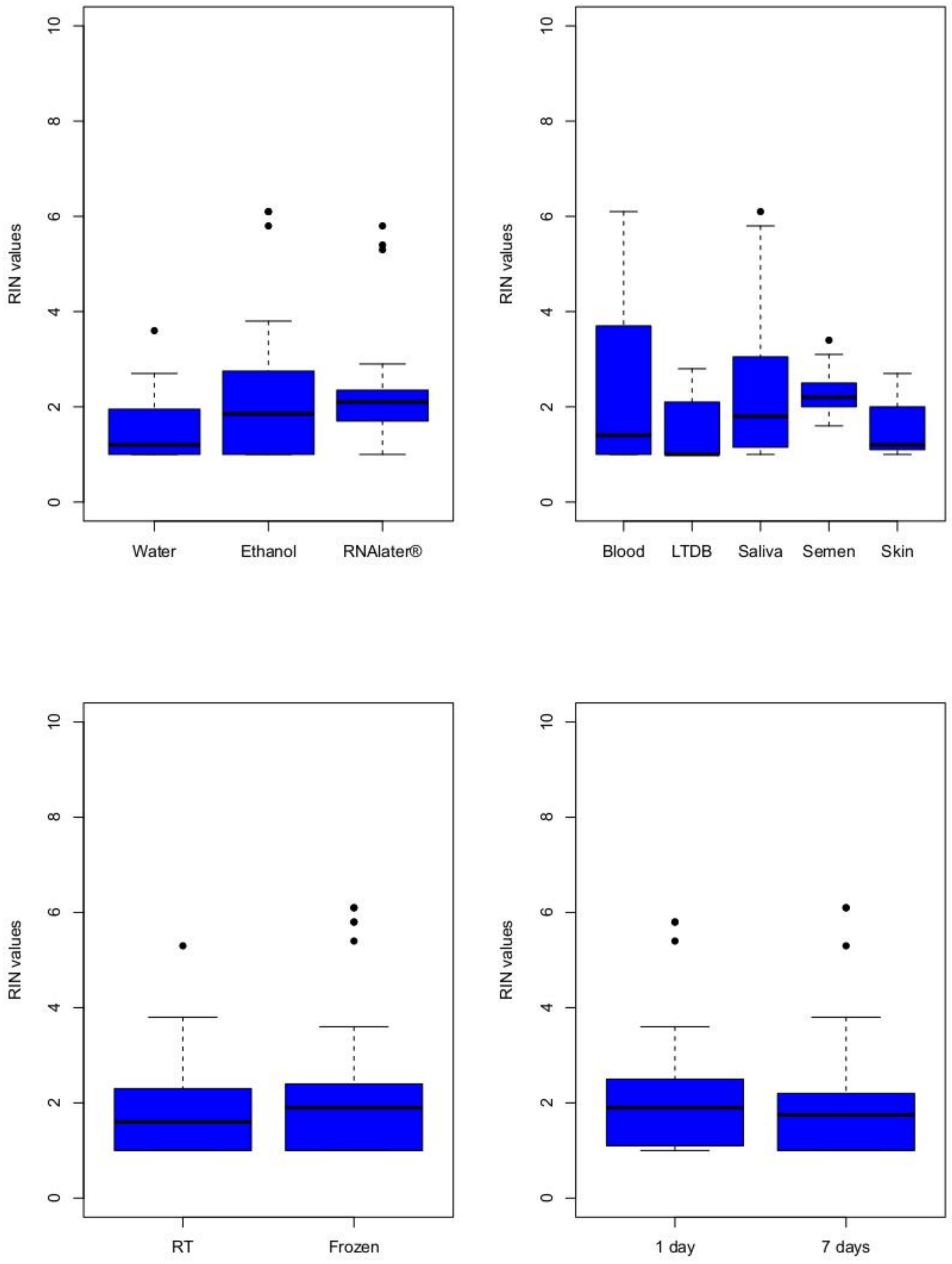

Figure S1. Box-and-whisker plots of the RIN values subdivided by moistening agent, tissue type (LTDB: luminol-treated diluted blood), storage temperature (RT: room temperature) and swab storage time before processing.

a)

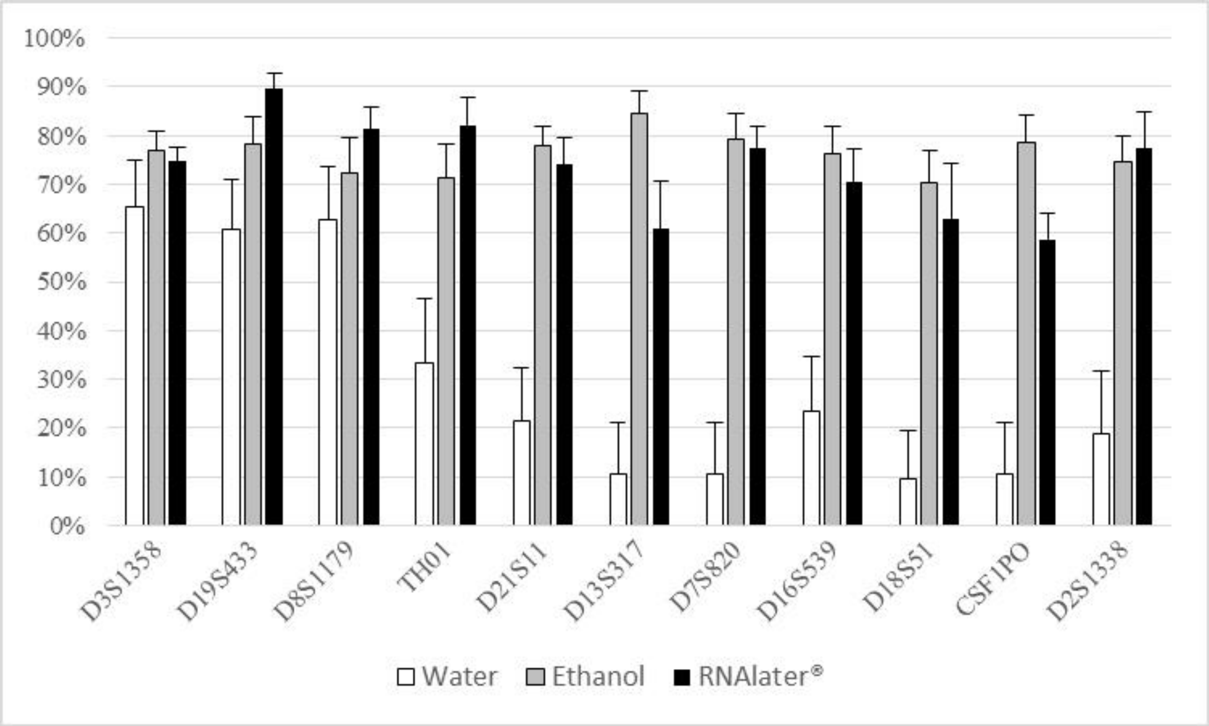

b)

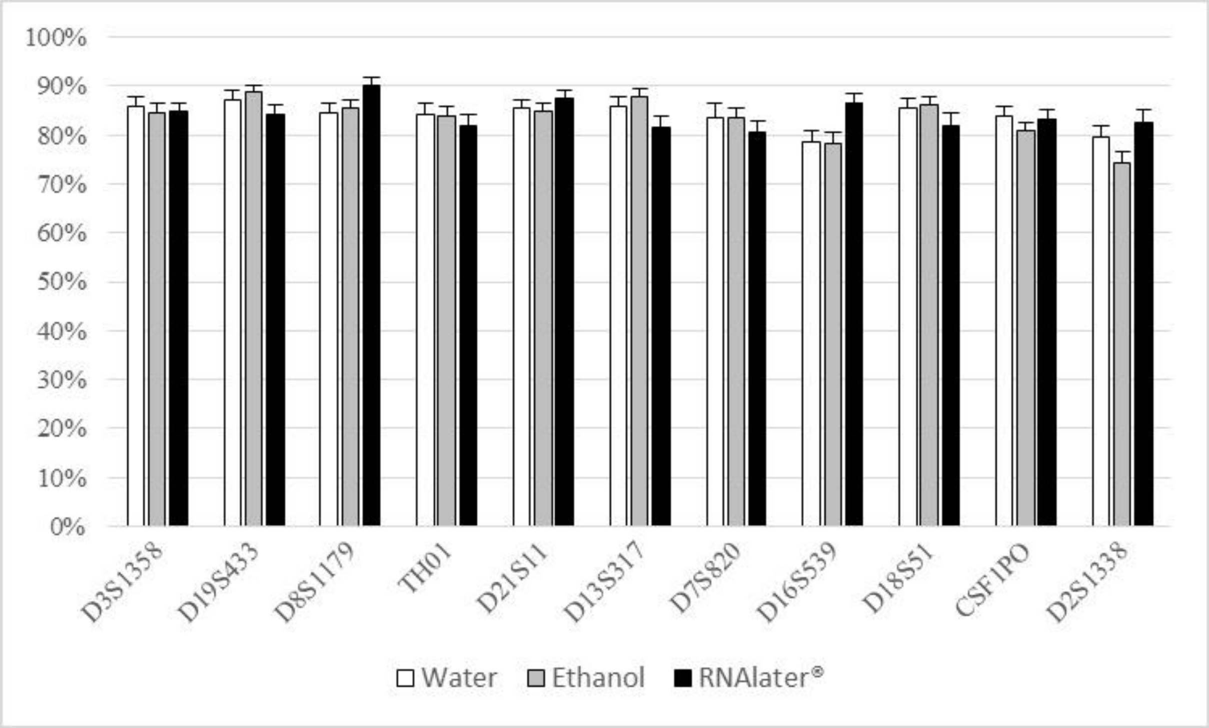

Figure S2. Average PHR in skin samples (a) and in tissue samples other than skin (b) treated with different moistening agents (whiskers indicate standard errors). STR markers with heterozygous genotype in the DNA profile of the donor are shown and listed according to amplicon size. PHR = 0% was assumed for loci with one of the two alleles below the analytical threshold (50 rfu).

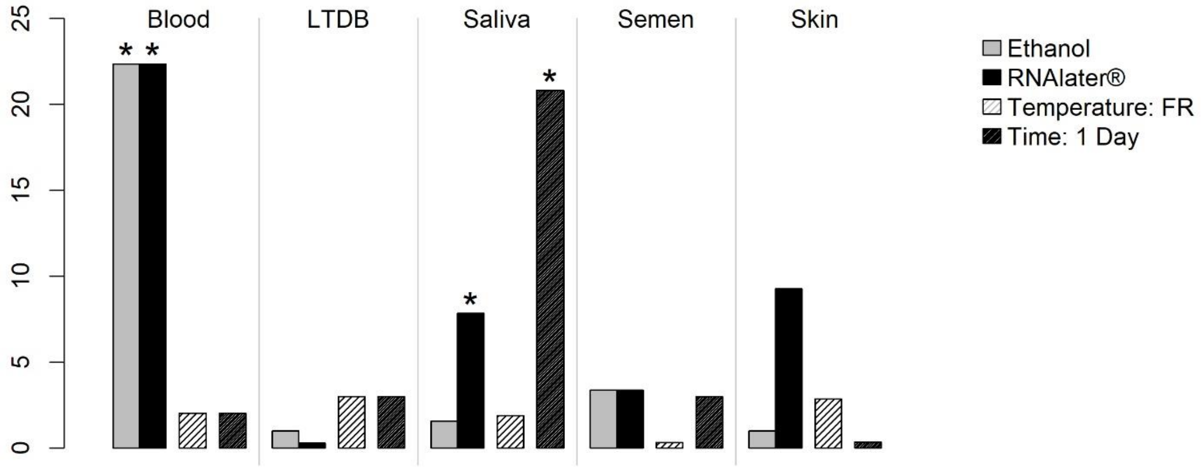

Figure S3. Odds ratios (ORs) reported on the y-axis indicate how much more likely it was to "observe" the expected tissues in the mRNA profiling experiments when alternative swabbing procedures were adopted instead of the standard procedure (i.e. swabs moistened with water and stored at room temperature for 7 days before extraction). Significant p-values are marked with an asterisk. LTDB: luminol-treated diluted blood.

Table S1. Generalized log-gamma regression coefficients represent the mean increase, or decrease, in DNA and RNA concentrations on a log scale due to the term, compared to the standard procedure (i.e. swabs moistened with water and stored at room temperature for 7 days before extraction).

Term	DNA concentration				RNA concentration			
	Coefficient β	lower 95% CI	upper 95% CI	p-value	Coefficient β	lower 95% CI	upper 95% CI	p-value
**Blood**								
Ethanol	-2.696	-3.453	-1.938	0.000	-1.765	-3.466	-0.093	0.015
RNAlater®	-1.196	-1.947	-0.445	0.005	0.046	-1.375	1.462	0.945
Frozen	-0.137	-0.747	0.474	0.660	-0.119	-1.235	1.000	0.826
1Day	-0.001	-0.613	0.613	1.000	1.650	0.273	2.997	0.006
**Luminol-treated diluted blood**								
Ethanol	-0.049	-0.419	0.322	0.799	1.133	0.489	1.774	0.002
RNAlater®	0.248	-0.123	0.619	0.204	3.711	3.088	4.332	0.000
Frozen	0.371	0.068	0.674	0.026	0.360	-0.146	0.869	0.176
1Day	-0.135	-0.438	0.169	0.393	0.200	-0.360	0.751	0.444
**Saliva**								
Ethanol	-0.522	-0.952	-0.091	0.027	0.135	-0.555	0.804	0.701
RNAlater®	0.119	-0.315	0.552	0.593	0.585	-0.110	1.267	0.119
Frozen	1.023	0.661	1.384	0.000	-0.693	-1.231	-0.150	0.024
1Day	0.152	-0.207	0.511	0.403	2.045	1.481	2.595	0.000
**Semen**								
Ethanol	-0.755	-1.177	-0.333	0.002	0.949	-0.018	1.897	0.058
RNAlater®	0.008	-0.417	0.433	0.972	1.772	0.732	2.814	0.002
Frozen	-0.254	-0.601	0.092	0.157	-0.076	-0.904	0.756	0.846
1Day	0.139	-0.204	0.483	0.430	-1.002	-1.892	-0.136	0.019
**Skin**								
Ethanol	1.779	0.606	2.955	0.004	-0.251	-0.895	0.385	0.443
RNAlater®	1.578	0.338	2.848	0.010	1.420	0.785	2.046	0.000
Frozen	-0.312	-1.351	0.698	0.521	0.066	-0.525	0.660	0.818
1Day	-0.679	-1.682	0.359	0.171	-0.433	-1.028	0.174	0.141

Table S2. Logistic regression ORs indicating the likelihood of "observing", rather than "not observing", the expected tissue in the mRNA profiling experiments due to the term, compared to the standard procedure (i.e. swabs moistened with water and stored at room temperature for 7 days before extraction).

Term	OR	lower 95% CI	upper 95% CI	p-value
**Blood**				
Ethanol	22.35	1.884	3288.25	0.01
RNAlater®	22.35	1.884	3288.25	0.01
Frozen	2.03	0.192	32.56	0.554
1 Day	2.03	0.192	32.56	0.554
**Luminol-treated diluted blood**				
Ethanol	1.000	0.005	195.918	1.000
RNAlater®	0.298	0.002	6.404	0.447
Frozen	3.009	0.160	448.967	0.469
1 Day	3.009	0.160	448.967	0.469
**Saliva**				
Ethanol	1.571	0.242	10.967	0.434
RNAlater®	7.857	1.020	102.436	0.048
Frozen	1.889	0.395	9.697	0.635
1Day	20.810	3.165	253.10	<0.001
**Semen**				
Ethanol	3.360	0.156	538.702	0.447
RNAlater®	3.360	0.156	538.702	0.447
Frozen	0.332	0.002	6.255	0.469
1 Day	3.009	0.160	448.967	0.469
**Skin**				
Ethanol	1.000	0.112	8.920	1.000
RNAlater®	9.273	0.526	1561.442	0.140
Frozen	2.849	0.367	36.234	0.325
1 Day	0.351	0.028	2.725	0.325

Table S3. Generalized log-gamma regression coefficients represent the mean increase, or decrease, in the peak height (rfu) of the tissue-specific mRNA markers and the housekeeping genes due to the term, compared to the standard procedure (i.e. swabs moistened with water and stored at room temperature for 7 days before extraction).

	**Tissue specific mRNA markers**				**Housekeeping genes**			
Term	Coefficient β	lower 95% CI	upper 95% CI	p-value	Coefficient β	lower 95% CI	upper 95% CI	p-value
**Blood**								
Ethanol	0.989	0.372	1.612	0.002	0.787	0.324	1.251	0.002
RNAlater®	1.279	0.665	1.900	<0.001	1.130	0.671	1.590	<0.001
Frozen	0.599	0.093	1.114	0.018	0.282	-0.094	0.659	0.140
1Day	0.293	-0.172	0.758	0.221	0.109	-0.255	0.475	0.557
**Luminol-treated diluted blood**								
Ethanol	-0.005	-0.285	0.274	0.970	-0.130	-0.430	0.170	0.405
RNAlater®	0.999	0.720	1.277	<0.001	0.955	0.653	1.258	<0.001
Frozen	0.102	-0.126	0.330	0.387	0.105	-0.140	0.349	0.411
1Day	0.187	-0.039	0.414	0.121	-0.140	-0.387	0.107	0.275
**Saliva**								
Ethanol	0.702	-0.118	1.515	0.084	0.828	0.163	1.486	0.020
RNAlater®	2.205	1.423	2.978	<0.001	1.647	0.928	2.361	<0.001
Frozen	0.144	-0.493	0.775	0.648	0.379	-0.155	0.912	0.163
1Day	2.746	2.099	3.388	<0.001	2.029	1.452	2.602	<0.001
**Semen**								
Ethanol	0.781	0.604	0.959	<0.001	0.924	0.762	1.087	<0.001
RNAlater®	0.905	0.727	1.082	<0.001	0.958	0.796	1.120	<0.001
Frozen	0.155	0.011	0.300	0.049	0.114	-0.018	0.247	0.107
1Day	-0.039	-0.184	0.106	0.606	0.015	-0.118	0.147	0.829
**Skin**								
Ethanol	0.451	-0.219	1.121	0.203	0.598	0.267	0.929	0.002
RNAlater®	0.870	0.200	1.541	0.020	0.610	0.279	0.279	0.002
Frozen	0.216	-0.331	0.763	0.449	0.172	-0.097	0.443	0.225
1Day	-1.083	-1.630	-0.535	0.001	-0.319	-0.589	-0.049	0.030
